# Supplementary material for: HIVprotI: an integrated web based platform for prediction and design of HIV proteins inhibitors
Source: J Cheminform. 2018 Mar 9;10:12. doi: 10.1186/s13321-018-0266-y (PMC5845081; doi:10.1186/s13321-018-0266-y)
Supplement: Supplementary file 2 — Additional file 2. Source code of HIVProtI web server. [file 13321_2018_266_MOESM2_ESM.zip › HIVprotI_Source-code/hivprotI/weka-3-6-6/doc/weka/classifiers/functions/neural/NeuralNode.html]

NeuralNode


---


|  |  |  |
| --- | --- | --- |
| **Overview** \| **Package** \| **Class** \| **Tree** \| **Deprecated** \| **Index** \| **Help** \| **Weka's home** | |  |
| **PREV CLASS**   **NEXT CLASS** | **FRAMES**    **NO FRAMES**     **All Classes** |  |
| SUMMARY: NESTED \| FIELD \| CONSTR \| METHOD | DETAIL: FIELD \| CONSTR \| METHOD |  |


---


## weka.classifiers.functions.neural Class NeuralNode

```
java.lang.Object
  weka.classifiers.functions.neural.NeuralConnection
      weka.classifiers.functions.neural.NeuralNode
```

**All Implemented Interfaces:**: java.io.Serializable, RevisionHandler

---

``` public class NeuralNode extends NeuralConnection ```

This class is used to represent a node in the neuralnet.

**Version:**
:   $Revision: 5403 $

**Author:**
:   Malcolm Ware (mfw4@cs.waikato.ac.nz)

**See Also:**: Serialized Form

---

**Field Summary**

**Fields inherited from class weka.classifiers.functions.neural.NeuralConnection**

`CONNECTED, INPUT, OUTPUT, PURE_INPUT, PURE_OUTPUT, UNCONNECTED`


**Constructor Summary**

`NeuralNode(java.lang.String id,
java.util.Random r,
NeuralMethod m)`


| **Method Summary** | |
| --- | --- |
| `double` | `errorValue(boolean calculate)`             Call this to get the error value of this unit. |
| `double[]` | `getChangeInWeights()`             call this function to get the chnage in weights array. |
| `NeuralMethod` | `getMethod()` |
| `java.lang.String` | `getRevision()`             Returns the revision string. |
| `double[]` | `getWeights()`             call this function to get the weights array. |
| `double` | `outputValue(boolean calculate)`             Call this to get the output value of this unit. |
| `void` | `removeAllInputs()`             This function will remove all the inputs to this unit. |
| `void` | `reset()`             Call this to reset the value and error for this unit, ready for the next run. |
| `void` | `restoreWeights()`             Call this to have the connection restore from the saved weights. |
| `void` | `saveWeights()`             Call this to have the connection save the current weights. |
| `void` | `setMethod(NeuralMethod m)`             Set how this node should operate (note that the neural method has no internal state, so the same object can be used by any number of nodes. |
| `void` | `updateWeights(double l, double m)`             Call this function to update the weight values at this unit. |
| `double` | `weightValue(int n)`             Call this to get the weight value on a particular connection. |

**Methods inherited from class weka.classifiers.functions.neural.NeuralConnection**

`connect, disconnect, drawHighlight, drawInputLines, drawNode, drawOutputLines, getId, getInputNums, getInputs, getNumInputs, getNumOutputs, getOutputNums, getOutputs, getType, getX, getY, onUnit, removeAllOutputs, setType, setX, setY`

**Methods inherited from class java.lang.Object**

`equals, getClass, hashCode, notify, notifyAll, toString, wait, wait, wait`

**Constructor Detail**

### NeuralNode

```
public NeuralNode(java.lang.String id,
                  java.util.Random r,
                  NeuralMethod m)
```

**Parameters:**: `id` - The string name for this node (used to id this node).: `r` - A random number generator used to generate initial weights.: `m` - The methods this node should use to update.


**Method Detail**

### setMethod

```
public void setMethod(NeuralMethod m)
```

:   Set how this node should operate (note that the neural method has no
    internal state, so the same object can be used by any number of nodes.

    :   **Parameters:**: `m` - The new method.

---


### getMethod

```
public NeuralMethod getMethod()
```

---


### outputValue

```
public double outputValue(boolean calculate)
```

:   Call this to get the output value of this unit.

    :   **Specified by:**: `outputValue` in class `NeuralConnection`
    :   **Parameters:**: `calculate` - True if the value should be calculated if it hasn't been already. **Returns:**: The output value, or NaN, if the value has not been calculated.

---


### errorValue

```
public double errorValue(boolean calculate)
```

:   Call this to get the error value of this unit.

    :   **Specified by:**: `errorValue` in class `NeuralConnection`
    :   **Parameters:**: `calculate` - True if the value should be calculated if it hasn't been already. **Returns:**: The error value, or NaN, if the value has not been calculated.

---


### reset

```
public void reset()
```

:   Call this to reset the value and error for this unit, ready for the next
    run. This will also call the reset function of all units that are
    connected as inputs to this one.
    This is also the time that the update for the listeners will be performed.

    :   **Specified by:**: `reset` in class `NeuralConnection`

---


### saveWeights

```
public void saveWeights()
```

:   Call this to have the connection save the current
    weights.

    :   **Specified by:**: `saveWeights` in class `NeuralConnection`

---


### restoreWeights

```
public void restoreWeights()
```

:   Call this to have the connection restore from the saved
    weights.

    :   **Specified by:**: `restoreWeights` in class `NeuralConnection`

---


### weightValue

```
public double weightValue(int n)
```

:   Call this to get the weight value on a particular connection.

    :   **Overrides:**: `weightValue` in class `NeuralConnection`
    :   **Parameters:**: `n` - The connection number to get the weight for, -1 if The threshold weight should be returned. **Returns:**: The value for the specified connection or if -1 then it should return the threshold value. If no value exists for the specified connection, NaN will be returned.

---


### getWeights

```
public double[] getWeights()
```

:   call this function to get the weights array.
    This will also allow the weights to be updated.

    :   **Returns:**: The weights array.

---


### getChangeInWeights

```
public double[] getChangeInWeights()
```

:   call this function to get the chnage in weights array.
    This will also allow the change in weights to be updated.

    :   **Returns:**: The change in weights array.

---


### updateWeights

```
public void updateWeights(double l,
                          double m)
```

:   Call this function to update the weight values at this unit.
    After the weights have been updated at this unit, All the
    input connections will then be called from this to have their
    weights updated.

    :   **Overrides:**: `updateWeights` in class `NeuralConnection`
    :   **Parameters:**: `l` - The learning rate to use.: `m` - The momentum to use.

---


### removeAllInputs

```
public void removeAllInputs()
```

:   This function will remove all the inputs to this unit.
    In doing so it will also terminate the connections at the other end.

    :   **Overrides:**: `removeAllInputs` in class `NeuralConnection`

---


### getRevision

```
public java.lang.String getRevision()
```

:   Returns the revision string.

    :   **Returns:**: the revision


---


|  |  |  |
| --- | --- | --- |
| **Overview** \| **Package** \| **Class** \| **Tree** \| **Deprecated** \| **Index** \| **Help** \| **Weka's home** | |  |
| **PREV CLASS**   **NEXT CLASS** | **FRAMES**    **NO FRAMES**     **All Classes** |  |
| SUMMARY: NESTED \| FIELD \| CONSTR \| METHOD | DETAIL: FIELD \| CONSTR \| METHOD |  |


---
